# Pannus Is the New Prepuce? Penile Cancer in a Buried Phallus

**DOI:** 10.1155/2015/403545

**Published:** 2015-09-13

**Authors:** Jared Manwaring, Srinivas Vourganti, Dmitriy Nikolavsky, Alfredo L. Valente, Timothy Byler

**Affiliations:** ^1^Department of Urology, Upstate Medical University, 750 East Adams Street, Syracuse, NY 13210, USA; ^2^Department of Pathology, Upstate Medical University, 750 East Adams Street, Syracuse, NY 13210, USA

## Abstract

Two males presented to our urology department with complaints of bleeding and malodor from buried phallus within a suprapubic fat pad. Although both men had neonatal circumcisions, advanced penile carcinoma was found in both men. Formal penectomies showed high grade, poorly differentiated squamous cell carcinoma invading the corporal bodies and urethra. Buried penis represents a difficulty in early detection of suspicious lesions but may also provide an environment susceptible to poor hygiene and subsequent chronic inflammation. Patients with buried penis may be at a higher risk for development of invasive penile cancer and may benefit from regular and thorough genital exams.

## 1. Introduction

We present two cases of men who presented with advanced penile carcinoma in the setting of a buried phallus. We describe the presentation, clinical course, and pathological findings and discuss the possible etiological agents involved in penile carcinoma.

## 2. Case Presentation

**Figure 1:**
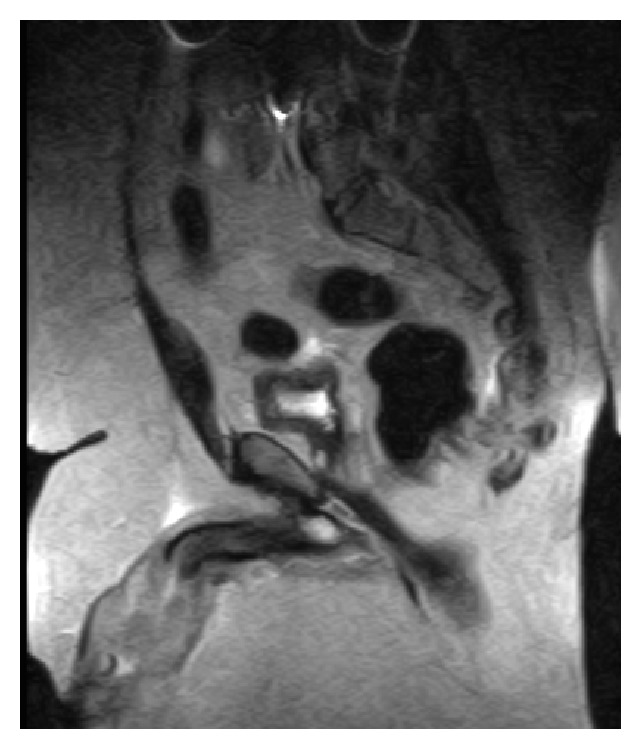
Coronal section of MRI showing the corporal bodies leading to a large mass buried within the suprapubic fat pad.

**Figure 2:**
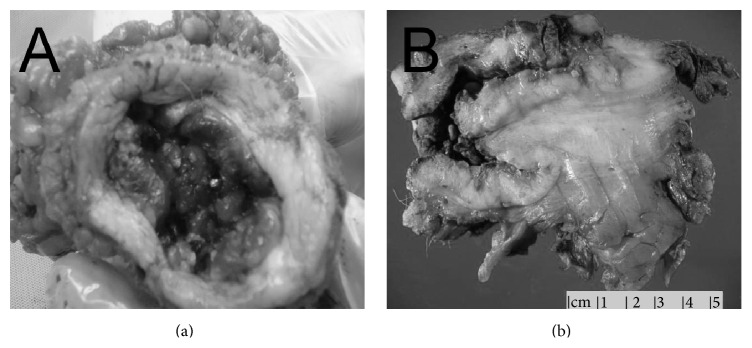
Gross specimens showing the buried phallus without identifiable structures (a) and a cross section showing the mass (b).

A 42-year-old Caucasian male with body mass index of 54 presented to outpatient clinic with 70-pound weight loss, hematuria, and purulent, malodorous discharge from the site of his buried penis. He was circumcised as an infant and denied any smoking history. The phallus was contained within a suprapubic fat pad and he had been sitting to void without seeing the phallus for several months. Although overall hygiene was adequate, the suprapubic cavity was impossible to maintain hygiene. Examination caused significant discomfort in attempt to visualize the phallus while in clinic prompting an examination under anesthesia. MRI revealed corporal bodies with a large mass replacing the glans penis and distal shaft (Figure 1). At the time of examination, the phallus was replaced by tumor and no identifiable structures were apparent (Figure 2). A suprapubic tube was placed and biopsies were taken. After appropriate discussion of treatment options, a total penectomy was performed. Pathologic examination of surgical specimens revealed high grade, poorly differentiated squamous cell carcinoma replacing the corporal bodies and urethra, pT3 with staining which was positive for p16. The lymph nodes were radiographically negative, but the patient chose to have radiotherapy to his lymph nodes rather than inguinal lymph node dissection. Despite negative surgical margins, he locally recurred with a cystic cavity within the suprapubic fat pad about 6 months after surgery. This was excised with wide margins and he is currently undergoing system chemotherapy.

**Figure 3:**
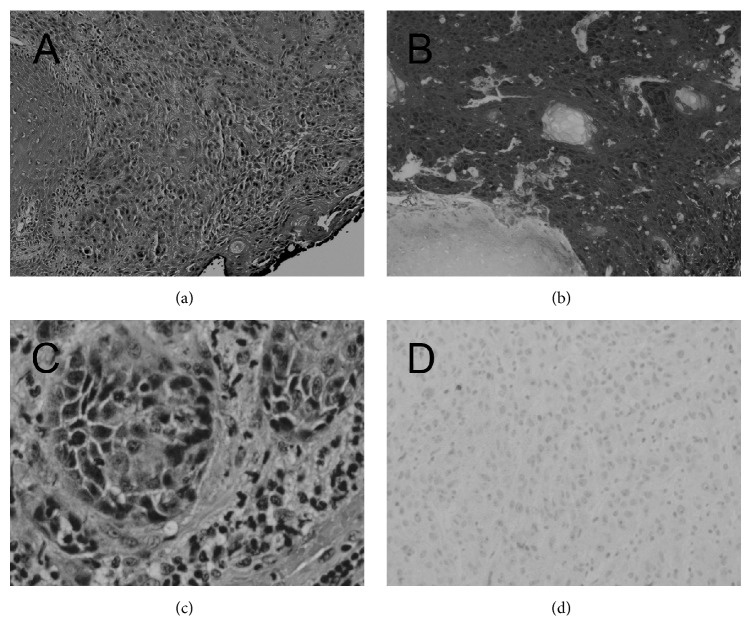
Histologic sections showing invasive squamous cell carcinoma of each patient (a) and (c) and immunohistochemistry of p16 with positive result (b) and negative result (d).

A 57-year-old Caucasian male with body mass index of 59 presented to the emergency department with complaints of bleeding and purulent discharge from the site of his buried penis. He was also circumcised as an infant and denied history of smoking. Once again, the phallus was contained within a suprapubic fat pad and was unable to be examined due to pain. He had also been sitting to void with a severely weakened stream and lack of phallus visualization for several months. Examination under anesthesia with biopsy was consistent with high grade invasive penile cancer. Total penectomy was then performed. Pathological examination showed moderately differentiated squamous cell carcinoma with corporal cavernosum invasion, pT3 with negative p16 staining. Representative sections are in Figure 3. Metastatic work-up including chest and abdominal imaging did not reveal any concerning lymph nodes or metastatic lesions. Prior to any adjunctive therapy, he had worsening pulmonary function and ultimately succumbed to pulmonary failure unrelated to the penile carcinogenesis.

## 3. Discussion

Given the rarity of penile carcinoma, these two cases may represent an enlarging demographic at risk that may need further consideration [1]. Although HPV was found on one of the surgical specimens, these men represent young, nonsmokers who were circumcised as neonates and therefore should be at low risk for penile cancer. We believe the buried phallus enhanced carcinogenesis and this represents a new group of patients that must be carefully examined and counseled regarding penile carcinoma.

Penile cancer was estimated to have 1640 new cases and cancer specific morality of 380 within the USA in 2014 [2]. Within the Swedish National Penile Cancer registry, the incidence has been stable and a high proportion of tumors were localized [3]. Classic risk factors include uncircumcised phallus, tobacco, psoriasis treatment with ultraviolet light, lichen sclerosis, sexually transmitted diseases including HIV and HPV, and poor hygiene. The median age of presentation in the United States is 68 [4].

As can be seen, both of these patients were younger than median and lacked many of the established risk factors, other than one patient having HPV incidentally found. The buried phallus is difficult to maintain hygiene and provides an area for a chronic inflammation and low grade infections. In a meta-analysis of circumcision performed by Larke [5], circumcision was shown to decrease penile cancer risk substantially. This was postulated to be mediated through the avoidance of phimosis and its associated inflammation [5]. The chronic inflammation of the uncircumcised phallus is replicated in the setting of buried phallus. This coupled with the poor hygiene and often chronic cavity infection may exaggerate the risk and induce neoplasia. Both men voided into the suprapubic cavity with inability to expose the phallus for hygiene creating an environment continually bathed in urine and prone to infectious processes.

Human papillomavirus (HPV) infection is a known risk factor for penile carcinogenesis and can be detected via staining with p16 [6]. The presence of the virus has not been shown to influence overall cancer outcome. In a retrospective study of 76 patients with penile tumors, authors found a positive HPV rate of 63% but this did not correlate with any pathological or clinical outcomes [7]. In a recent meta-analysis of European general population males, it was estimated that HPV positivity ranged between 12 and 30% [8]. Given our patient's young age at only 42 years, it is possible that the HPV helped accelerate his neoplastic process. Given that almost one-third of the population was positive for high risk HPV [8], it is more likely that the environment of the buried phallus, chronic inflammation, and poor hygiene were the initiating factors in his carcinogenesis.

Obesity has been a growing problem now affecting over 1/3 of US adults and 17% of the youth [9]. This has many health correlates well established including hypertension, diabetes, and heart disease and can lead to a buried phallus. Though obesity negatively affects other cancers, retrospective studies have not shown that BMI in itself correlates with penile cancer or penile cancer survival [10]. Males with a buried phallus suffer from failure of early detection and tumor surveillance in addition to the possibility of increased risk of neoplasia. Men generally recognize smaller lesions and come to physician attention before these advanced stages but the buried phallus prevents awareness of the lesion until tumor progression results in hematuria or necrosis. This argues for consistent genital exams even when it may be uncomfortable for the patient and physician emphasis on self-examination.

## 4. Conclusion

Penile cancer in the setting of buried phallus represents both an error of early detection and a possible increased risk. Patients and their physicians should routinely examine the buried phallus and evaluate further as indicated.
